# Hybrid Enhancement of Surface-Enhanced Raman Scattering Using Few-Layer MoS_2_ Decorated with Au Nanoparticles on Si Nanosquare Holes

**DOI:** 10.3390/nano12050786

**Published:** 2022-02-25

**Authors:** Tsung-Shine Ko, Yen-Lun Chen

**Affiliations:** Department of Electronic Engineering, National Changhua University of Education, No. 2, Shi-Da Road, Changhua 50074, Taiwan; seed120x02@gmail.com

**Keywords:** SERS, MoS_2_, Au nanoparticle, nanostructure

## Abstract

By combining the excellent biocompatibility of molybdenum disulfide (MoS_2_), excellent surface-enhanced Raman scattering (SERS) activity of Au nanoparticles (Au NPs), and large surface area of Si nanosquare holes (NSHs), a structure in which MoS_2_ is decorated with Au NPs on Si NSHs, was proposed for SERS applications. The NSH structure fabricated by e-beam lithography possessed 500 nm of squares and a depth of approximately 90 nm. Consequently, a few-layer MoS_2_ thin films (2–4 layers) were grown by the sulfurization of the MoO_3_ thin film deposited on Si NSHs. SERS measurements indicated that MoS_2_ decorated with Au NPs/Si NSHs provided an extremely low limit of detection (ca. 10^−11^ M) for R6G, with a high enhancement factor (4.54 × 10^9^) relative to normal Raman spectroscopy. Our results revealed that a large surface area of the NSH structure would probably absorb more R6G molecules and generate more excitons through charge transfer, further leading to the improvement of the chemical mechanism (CM) effect between MoS_2_ and R6G. Meanwhile, the electromagnetic mechanism (EM) produced by Au NPs effectively enhances SERS signals. The mechanism of the SERS enhancement in the structure is described and discussed in detail. By combining the hybrid effects of both CM and EM to obtain a highly efficient SERS performance, MoS_2_ decorated with Au NPs/Si NSHs is expected to become a new type of SERS substrate for biomedical detection.

## 1. Introduction

Surface-enhanced Raman scattering (SERS) has become a powerful method for the detection of biomolecules, owing to its many excellent advantages, including improvement of the Raman scattering efficiency, high sensitivity at the single-molecule level, and high accuracy in biomedical detection [1,2,3]. In the last few years, various SERS substrates, such as metallic structures [4], compound semiconductors [5,6], and two-dimensional (2D) materials [7,8] have garnered an increasing interest. Among SERS substrates, the main mechanisms for SERS enhancement can be divided into electromagnetic (EM) and chemical (CM) mechanisms [9,10]. The EM effect is attributed to the local electromagnetic field enhancement at the metal surface owing to the surface plasmon polaritons, yielding an excellent SERS enhancement [11]. Noble metals, such as Au and Ag, are widely used as SERS substrates due to the existence of EMs. However, the main disadvantages of using noble metals as SERS substrates are their high cost, poor stability, and strong carbonization effects [12,13,14]. Moreover, the SERS enhancement of the CM effect owing to a charge transfer (CT) between the probe molecule and semiconductor is relatively weaker than that of the EM effect. Instead, semiconductor SERS substrates offer a high stability and biocompatibility [15]. Therefore, a hybrid SERS substrate that combines the advantages of the EM and CM effects should be developed and investigated.

Molybdenum disulfide (MoS_2_) is a 2D material that has a significant biocompatibility with a flat and well-bonded surface, leading to the formation of CT states in close proximity to molecules, which considerably enhances the SERS signal [16]. Recently, some studies have investigated the use of MoS_2_ and various nanometal particles as hybrid SERS substrates. Jiang et al. synthesized MoS_2_/Ag nanoparticles (Ag NPs) on a pyramidal silicon structure to obtain a high sensitivity with an enhancement factor (EF) of 9.55 × 10^6^ for Rhodamine 6G (R6G) as a SERS probe [17]. Dou et al. utilized Ag NP-decorated mesh-like MoS_2_ hierarchical nanostructures, which possess a high sensitivity, reasonable detection capability, and satisfactory reproducibility for trace malachite green in flowing water [18]. Chen et al. used a thermal decomposition method to synthesize a few-layer MoS_2_ on Ag NP surfaces to form a hybrid system that reached a limit of detection (LOD) of 10^−9^ M for R6G [19]. According to these studies, using hybrid structures as SERS substrates could further enhance sensitivity. However, studies on the individual effects of either EM or CM considering these hybrid structures are still limited. In addition, the relationship between EM and CM is not yet clear.

In this study, we propose a few-layer MoS_2_ decorated with Au nanoparticles (Au NPs) on a Si nanosquare hole (NSH) structure as a new type of SERS substrate. The Si NSH structure was fabricated using a standard semiconductor process. After the MoO_3_ layer was coated using the thermal evaporation technique, a few-layer MoS_2_ thin film was grown by sulfurization. After decorating Au NPs on the MoS_2_/Si NSHs sample as a SERS substrate, we further used scanning electron microscopy (SEM) and Raman spectroscopy to analyze the material properties of the structure. Atomic force microscopy (AFM) was performed to analyze the depth of Si NSH. Our SERS substrate provided an EF of 4.54 × 10^9^ and an LOD of approximately 10^−11^ M for R6G as a test molecule, revealing an excellent SERS performance. In addition, the SERS performance using different types of substrates, including planar Si substrate, planar MoS_2_/Si, MoS_2_/Si NSHs, and Au NPs/Si NSHs, was investigated for comparison purposes. In this study, we also examined individual effects of both EM and CM to provide a detailed discussion.

## 2. Materials and Methods

### 2.1. Fabrication of MoS_2_ Decorated with Au NPs/Si NSHs

**Figure 1:**
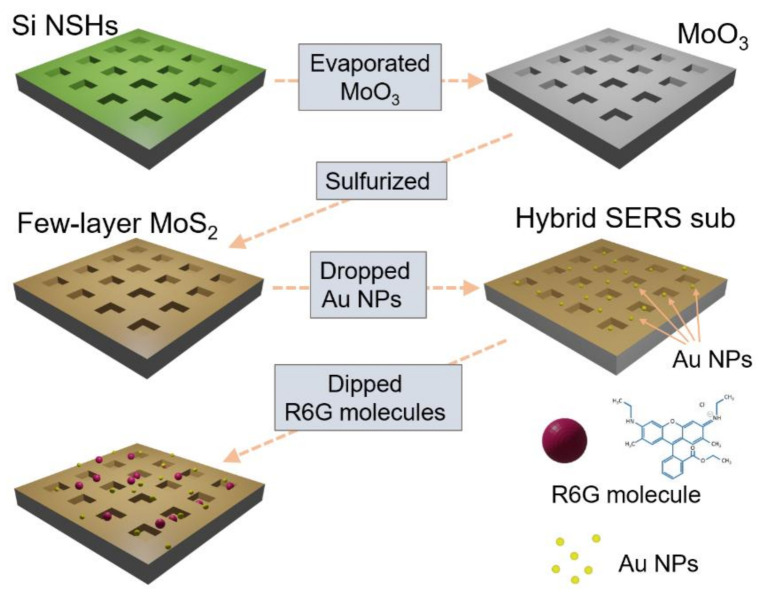
Schematic illustration of the fabrication of MoS_2_ decorated with Au NPs on Si NSHs.

Boron-doped single-crystal Si wafers were cleaned using a standard cleaning process [20]. A positive photoresist (PR) was uniformly coated on Si wafers using a spin coater. Subsequently, we utilized a direct write e-beam lithography system (ELIONIX ELS7500-EX, Tokyo, Japan) to determine square patterns with side lengths of approximately 500 nm, and then fabricated the samples by using the following procedure. We used Cl_2_/HBr plasma to etch Si; the etch depth was adjusted by controlling the etching time. Eventually, the PR layer was removed using an O_2_ plasma. In addition to the fabrication of Si NSHs for the growth of MoS_2_, bare Si wafers were prepared as substrates to obtain planar MoS_2_ thin films for comparison. In this study, all MoS_2_ structures were obtained in a furnace by sulfurizing MoO_3_ thin films coated on various substrates. The MoO_3_ method and subsequent sulfurization for MoS_2_ growth were reported in our previous study [21]. We maintained the same parameters in this study, except for decreasing the deposition time of MoO_3_ to 5 s to obtain a few-layer MoS_2_ structure. The Au NP solution (0.8 mM) was produced by the chemical reduction of gold chloride tetrahydrate (HAuCl_4_) with sodium citrate [22]. A 30 μL Au NPs solution was pipetted out and dripped onto various substrates. Subsequently, the samples were heated on a hot plate at 70 °C for 5 min under ambient conditions until the Au NP droplets were dried. R6G (99%, ACROS Organics, Geel, Belgium) was dissolved in water (at concentrations from 10^–2^ to 10^–12^ M) and dip-coated onto substrates, including the planar Si substrate, planar MoS_2_/Si, MoS_2_/Si NSHs, Au NPs/Si NSHs, and MoS_2_ decorated with Au NPs on Si NSHs. The samples were dried on a hot plate at 70 °C for 5 min under ambient conditions. A schematic flowchart is shown in Figure 1.

### 2.2. Characterizations

SEM (FEI Helios 1200+, Hillsboro, OR, United States) revealed the morphologies and nanostructures of the as-grown samples. An AFM scan (Veeco DI-3100, Plainview, NY, United States) was used to examine the depth of Si NSH. The Raman spectra and SERS analyses were preformed using a confocal Raman microscopy system (Nanofinder 30, Tokyo Instruments, Tokyo, Japan) with excitation from a He–Ne laser (laser power: 0.1 mW for as-grown samples; 0.3 mW for the reference substrate). The laser spot size was focused at 3 μm for the Raman spectra by adjusting the position of the microscope objective with a magnification of 100× and a numerical aperture of 0.9. The acquisition time for laser measurements was set to 20 s. The grating in the spectrometer was 300 lines/mm. The charge-coupled device mounted on the spectrometer was cooled to approximately −50 °C, using a thermoelectric cooling chip to minimize noises during the measurement. Before the measurement, the spectra were calibrated using the position of the Si peak (520 cm^–1^) from the bulk Si substrate.

## 3. Results and Discussion

**Figure 2:**
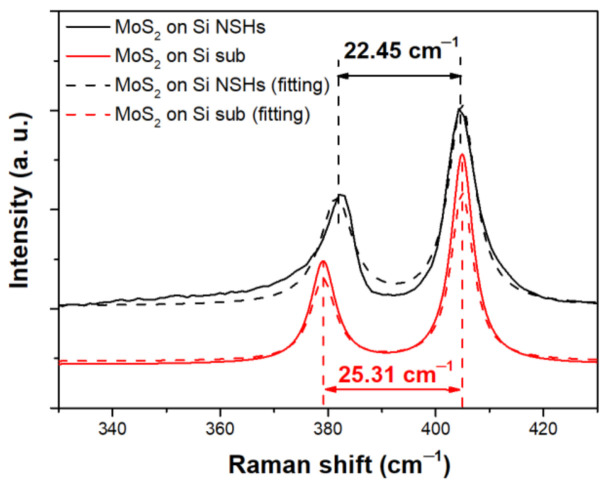
Raman spectra and corresponding Gaussian fitting results of few-layer MoS_2_ decorated with Au NPs/Si NSHs.

We used the Raman spectroscopy to analyze molecular vibration modes of the MoS_2_ thin films formed on the planar Si and Si NSH substrates after sulfurization. The Raman spectra and corresponding Gaussian fitting results for both the planar MoS_2_ and MoS_2_/Si NSHs are shown in Figure 2, which presents the typical E^1^_2g_ and A^1^_g_ vibration mode peaks of MoS_2_ for both samples, indicating that the MoO_3_ layer was successfully sulfurized into MoS_2_. Our Gaussian fitting results revealed that the difference in wavenumber between the two peaks was approximately 25.31 cm^−1^ for the planar MoS_2_ and 22.45 cm^−1^ for MoS_2_/Si NSHs. Since the difference in wavenumber between the E^1^_2g_ and A^1^_g_ modes could reflect the number of layers for MoS_2_ in terms of a few-layer structure [23,24], we suggested that the thickness of the MoS_2_ coated on the lateral wall and basin surfaces into the NSHs would be probably two to three layers, and that on the platform would be approximately four layers. The thickness range of two to four layers for the few-layer MoS_2_ structure provides a direct energy bandgap for excitons (electrons and electron holes), which is beneficial to enhance the SERS through the CT mechanism.

**Figure 3:**
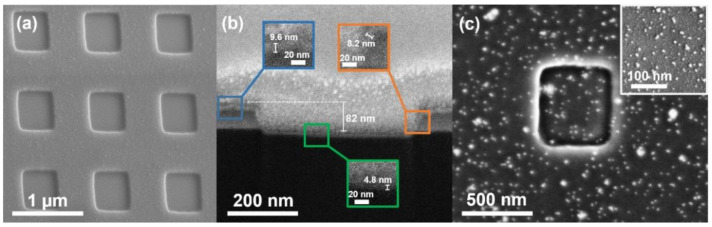
(**a**) Top-view and (**b**) cross-section SEM images of few-layer MoS_2_ /Si NSHs. The inserted images are enlarged from the corresponding areas of the platform, lateral wall and basin. (**c**) Top-view SEM image of few-layer MoS_2_/Si NSHs after spreading Au NPs. The inset is the top view amplified SEM image of the platform area.

The top-view and cross-sectional SEM images of MoS_2_/Si NSHs are shown in Figure 3a,b. Both SEM images revealed that the square side length and hole depth for the NSH structure is approximately 500 and 82 nm, respectively. The basin length was less than 500 nm; thus, we could infer that this cross-section was not along the central axis; however, it was close to the edge of the hole. To obtain the correct hole depth, we performed AFM to obtain the cross-sectional profile of the hole, as shown in Figure S1. The AFM image reveals that the correct depth is approximately 90 nm, which provides about 1.7 times surface area in comparison to the planar surface. In addition, we observed the thicknesses of the MoS_2_ thin film is approximately 9.6 nm on the platform, 8.2 nm on the lateral wall, and 4.8 nm on the basin, as shown in inset SEM images of Figure 3b. The MoS_2_ thicknesses obtained by SEM were thicker than those measured by the Raman spectroscopy, and we believe that the Raman spectroscopy results were still reasonable since an image vibration issue under the SEM inspection could cause a slightly larger corresponding thicknesses of MoS_2_ in the SEM images. The cross-sectional SEM image could not reveal the overall thickness of MoS_2_ on each facet. The thinner few-layer MoS_2_ was difficult to observe since the few-layer MoS_2_ was not a continuous thin film, and the interface between layers was low. This is due to the thicknesses of the MoS_2_ formed on the lateral and basin surfaces being thinner than those on the platform, which was probably owing to the lower efficiency of the gas reaction within the NSH area. Figure 3c shows the top-view SEM image after spreading Au NPs on the MoS_2_/Si NSHs, where we observed that Au NPs were uniformly distributed on either the platform or basin area of the sample. Only a few Au NPs aggregated into large sizes. The inserted SEM image reveals that the Au NP size is mainly in the range of 5–10 nm.

**Figure 4:**
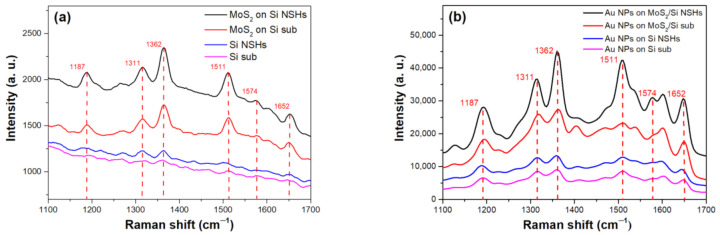
(**a**) Before and (**b**) after spreading Au NPs, SERS signals of R6G molecules (10^–6^ M) on planar Si, Si NSHs, few-layer MoS_2_/Si, and few-layer MoS_2_/Si NSHs substrates.

We dropped the R6G solution (~10^−6^ M) onto the planar Si substrate, Si NSHs, planar MoS_2_/Si, and MoS_2_/Si NSHs for SERS measurements to examine the effect of using the Si NSH array. Figure 4a shows the SERS signals of various samples obtained using the Raman spectroscopy for R6G detection. The planar Si substrate exhibited the lowest SERS intensity. The signal measured using Si NSHs as a SERS substrate was slightly enhanced compared to that of the planar Si. After the few-layer MoS_2_ was formed on the Si and Si NSHs, SERS signals of R6G were apparently enhanced owing to exciton resonance in the CT mechanism between MoS_2_ and R6G [25]. The peak intensity of using MoS_2_/Si NSHs was 1.36 times that of using the planar MoS_2_ thin film and 2.09 times that of using the Si substrate for a peak of 1362 cm^−1^. In comparison to the planar substrates, the NSH array had a larger surface area to improve more dye adsorption and generate more excitons through CT, leading to a higher SERS intensity. According to the above description of the Raman results, as shown in Figure 2, the average number of MoS_2_ layers in the NSH array could be less than that on the planar substrate. Mak et al. reported that an indirect transition between the conduction band at the Λ point (midpoint along Γ-K) and valence band at the Γ point in the MoS_2_ band diagram was gradually triggered more efficiently with an increase in the number of layers [26]. Here, we suggested that the slightly stronger SERS enhancement for the MoS_2_/Si NSHs than that of the planar MoS_2_, was due to the MoS_2_ having fewer layers on the Si NSHs structure, producing a higher intermolecular CT yield owing to the low opportunity of the indirect transition. Furthermore, differences in our SERS results between MoS_2_ and Si were worth discussing. Unlike Si, which was an indirect semiconductor, the few-layer MoS_2_ (2–4 layers) structure still provided a direct bandgap between the conduction and valence bands at the same K point for electrons, which was beneficial for the CT process to produce more excitons when the sample was under laser irradiation [26,27]. Moreover, a hexagonal atomic orientation with close proximity to the probe molecules in MoS_2_ thin films was an advantage for the SERS enhancement [16]. Consequently, we applied the same procedure to investigate the effect of using Au NPs on various substrates to enhance the SERS. Figure 4b shows the SERS results. After the dispersion of Au NPs, we observed that Raman signals of R6G molecules on various substrates were dramatically enhanced due to the formation of hot spots where Au NPs provided an electromagnetic field and more active sites for R6G molecules [28]. All modes of R6G exhibited the same trend in terms of the SERS intensity when measured on various substrates, according to the results in Figure 4a, indicating that the few-layer MoS_2_ and Si NSHs individually contributed to the SERS enhancement, even though the substrates were decorated with Au NPs. In order to confirm reproducibility and reliability for SERS results of using the few-layer MoS_2_/Si NSHs before and after spreading Au NPs for R6G detection, we randomly measured SERS signals of the R6G molecules (10^−6^ M) from the different positions on both samples, which are shown in Figure S2a,b. Obviously, the SERS results at different positions revealed similar profiles of SERS signal intensity, demonstrating that using the few-layer MoS_2_/Si NSHs before and after spreading Au NPs as SERS substrates have good reproducibility and reliability for R6G detection under laser spot size of ~3 μm condition.

**Figure 5:**
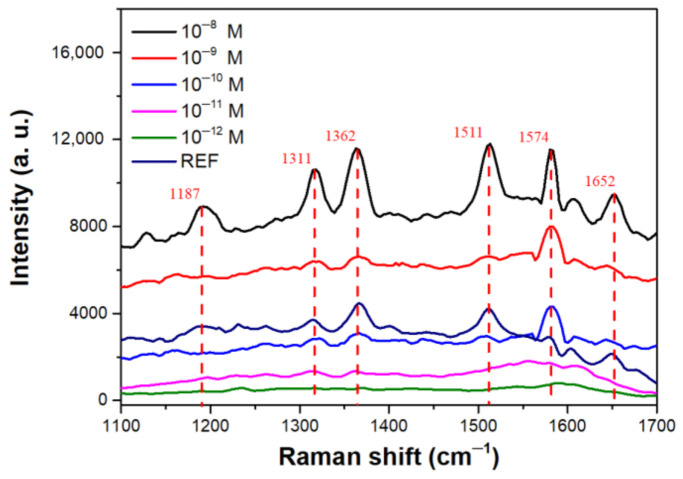
SERS signals of solutions of R6G at concentrations from 10^–8^ to 10^–12^ M on the few-layer MoS_2_ decorated with Au NPs on Si NSHs substrates. The curve REF is the SERS data for the planar Si in the presence of 10^–2^ M R6G, for comparison.

The SERS results obtained using various substrates indicated that the MoS_2_ decorated with Au NPs/Si NSHs structure yielded the best SERS enhancement for the detection of R6G. We further used solutions of R6G at concentrations from 10^−8^ to 10^−12^ M for dipping the MoS_2_ decorated with Au NPs/Si NSHs structure and a R6G solution at a concentration of 10^−2^ M for dipping flat Si as a reference substrate to determine the SERS efficiency. Figure 5 presents the results of SERS analyses of various samples. Intensities of the signals for vibrational modes of R6G decreased with a decreasing concentration of R6G from 10^−8^ to 10^−12^ M. The main 1362 and 1511 cm^−^^1^ modes remained distinguishable when the R6G solution with a concentration of 10^−11^ M was applied. All modes of R6G disappeared when the R6G concentration was 10^−12^ M. Therefore, LOD for R6G when using the MoS_2_ decorated with Au NPs/Si NSHs was approximately 10^−11^ M in our case. We selected a strong mode at 1362 cm^−1^ representing C-C stretching in the xanthene ring as a reference for the calculation of EF since it was intense and isolated from interference with nearby signals [29]. We compared the intensity of the peak at 1362 cm^−1^ for Au NPs/MoS_2_/Si NSHs with that of the reference substrate, with the relative EF calculated as follows [30]:

(1) $$\mathrm{EF}=\frac{I_{\mathrm{SERS}}}{I_{\mathrm{REF}}}\times \frac{N_{\mathrm{REF}}}{N_{\mathrm{SERS}}},$$

where *I* and *N* are the Raman spectral intensity and number of molecules, respectively; SERS and REF represent the values obtained from Au NPs/MoS_2_/Si NSHs and the reference sample of the flat Si substrate, respectively. Moreover, laser power was increased from 0.1 to 0.3 mW for the reference substrate to obtain distinguishable signals. Since the laser spot size and exposure time were identical for all measurements, the value of *N*_REF_/*N*_SERS_ was the same as the R6G concentration ratio for both samples. The estimated value of EF for the peak at 1362 cm^−1^ was found to be approximately 4.54 × 10^9^ for Au NPs/MoS_2_/Si NSHs. In addition, we carried out the same SERS experiments for the MoS_2_/Si NSHs and Au NPs/Si NSHs structures to obtain the corresponding EF and LOD results, and thus clarify the difference in the contribution of MoS_2_ (CM) and Au NPs (EM) to the SERS enhancement. The EF and LOD results for various substrates are summarized in Table 1, which indicates that the MoS_2_/Si NSH structure through the CM provided an LOD of ca. 10^−6^ M for R6G, with an EF of 7.86 × 10^4^. Furthermore, Au NP/Si NSHs obtained an LOD of ca. 10^−8^ M and an EF of 7.64 × 10^6^. Therefore, our SERS results demonstrated that using a MoS_2_ decorated with Au NPs/Si NSHs could combine hybrid effects of CT and EM to achieve a highly efficient SERS performance.

**Figure 6:**
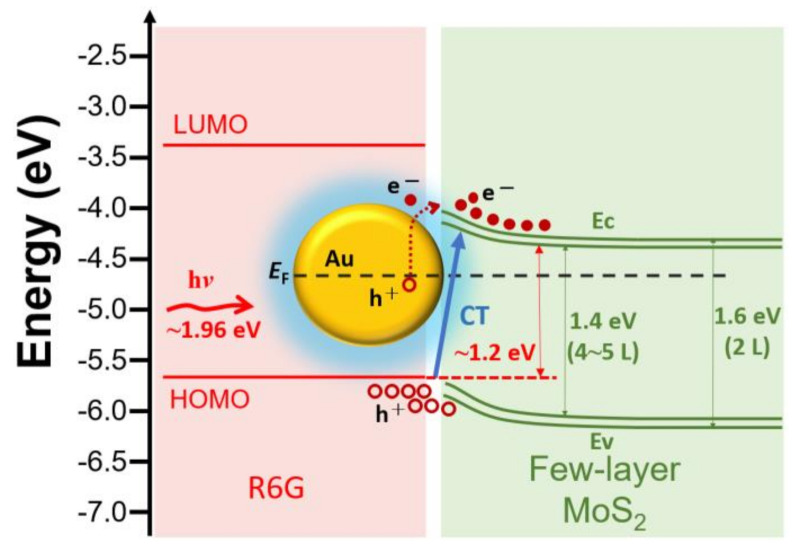
Schematic diagram of hybrid effects of both EM and CM between R6G molecules and few-layer MoS_2_ decorated with Au NPs on Si NSHs.

A schematic of R6G on the Au NPs/MoS_2_/Si NSHs structure is presented in Figure 6. In our case, R6G molecules contacted not only Au NPs but also the few-layer MoS_2_. Here, we discussed the band diagram of the hybrid substrate in two parts: (i) R6G/MoS_2_, and (ii) Au NPs/MoS_2_ interfaces. (i) The SERS mechanism for the molecule-semiconductor system had been demonstrated to be CM due to the CT process at the interface between the molecule and semiconductor [25,31,32]. When the sample was irradiated with a laser (~1.96 eV), electrons in the highest occupied molecular orbital (HOMO) of R6G molecules absorbed enough light energy to then jump to the adjacent conduction band of the few-layer MoS_2_ to form electron-hole pairs, that is, excitons. Meanwhile, electrons in the valence bands of the few-layer MoS_2_ and underneath Si NSHs also had the chance to jump to the corresponding conduction band. The excitons generated from Si NSHs can be neglected in our case since the existence of native silicon oxide films and extremely low enhancement of R6G using Si NSHs as a SERS substrate. The energy difference between the lowest unoccupied molecular orbital (LUMO) of R6G and the conduction band of the few-layer MoS_2_ formed a barrier to aggregate free electrons on the MoS_2_ side. The same explanation could be easily adopted to describe the aggregation of electron holes. Moreover, the energy difference between the LUMO and HOMO for R6G was approximately 2.3 eV, which was extremely high for electrons in HOMO to jump to LUMO. (ii) The Au NPs/MoS_2_ interface was a metal-semiconductor system. We assumed that the few-layer MoS_2_ was intrinsic and had no Fermi level pinning effect at the interface. A Schottky barrier exists at the junction interface between Au and MoS_2_. Electrons in Au NPs would overcome the Schottky barrier under the laser illumination; subsequently, they are transferred from Au to the conduction band of MoS_2_. The plasmonic effect of Au NPs produced an enhanced local electromagnetic field near the metal surface interacting with R6G molecules [33,34], leading to an increase in the atomic oscillation strength. The surroundings of Au NPs would form many active sites. Therefore, Au NPs in the structure are essential in inducing EM and contribute to the SERS enhancement. In addition, the aggregation of excitons or the main CT path in this structure primarily occurred at the interface between R6G and MoS_2_, which was also significantly influenced by the local electromagnetic field from Au NPs. Several resonances in the surface plasmons, excitons, and CT are strengthened by the local electromagnetic field, enhancing the SERS. Therefore, using the MoS_2_ decorated with Au NPs/Si NSHs as an effective SERS substrate could combine both the CM and EM effects to enhance SERS for use in biomedical detection.

## 4. Conclusions

We fabricated a few-layer MoS_2_ decorated with Au NPs on Si NSHs as a SERS substrate, which offers hybrid effects of both EM and CM. The corresponding SERS performances of both EM and CM, in terms of our SERS substrate, were obtained and compared. SERS measurements indicated that the few-layer MoS_2_ decorated with Au NPs on Si NSHs was a highly efficient SERS substrate, with a high EF of 4.54 × 10^9^ and an LOD of approximately 10^−11^ M for the detection of R6G. We suggested that the local electromagnetic field from Au NPs could magnify several resonances in the surface plasmons, excitons, and CT, leading to a SERS enhancement. Our experimental results demonstrated that a few-layer MoS_2_ decorated with Au NPs on Si NSHs is a potential SERS substrate for sensing probe molecules with a high efficiency and sensitivity.

## Figures and Tables

**Table 1:** The EF and LOD values obtained by using MoS_2_/Si NSHs, Au NPs/Si NSHs and MoS_2_ decorated with Au NPs on Si NSHs as SERS substrates.

Structures	EF	LOD (M)
MoS_2_/Si NSHs	7.86 × 10^4^	10^−6^
Au NPs/Si NSHs	7.64 × 10^6^	10^−8^
Au NPs on MoS_2_/Si NSHs	4.54 × 10^9^	10^−11^

## Data Availability

Data is contained within the article or supplementary material.

## Supplementary Materials

The following are available online at https://www.mdpi.com/article/10.3390/nano12050786/s1, Figure S1: AFM image and cross-sectional profiles along blue and red lines of few-layer MoS_2_ /Si NSHs; Figure S2: (a) Before and (b) after spreading Au NPs, SERS signals of R6G molecules (10^−6^ M) from randomly different positions 1–3 on few-layer MoS_2_/Si NSHs.
