# Supplementary material for: Hybrid Enhancement of Surface-Enhanced Raman Scattering Using Few-Layer MoS2 Decorated with Au Nanoparticles on Si Nanosquare Holes
Source: Nanomaterials (Basel). 2022 Feb 25;12(5):786. doi: 10.3390/nano12050786 (PMC8912446; doi:10.3390/nano12050786)
Supplement: Supplementary file 1 [file nanomaterials-12-00786-s001.zip › nanomaterials-1597635-supplementary.pdf]

nanomaterials

MDPI

Supplementary Materials

Hybrid Enhancement of Surface-Enhanced Raman Scattering Using Few-Layer MoS_2 Decorated with Au Nanoparticles on Si Nanosquare Holes

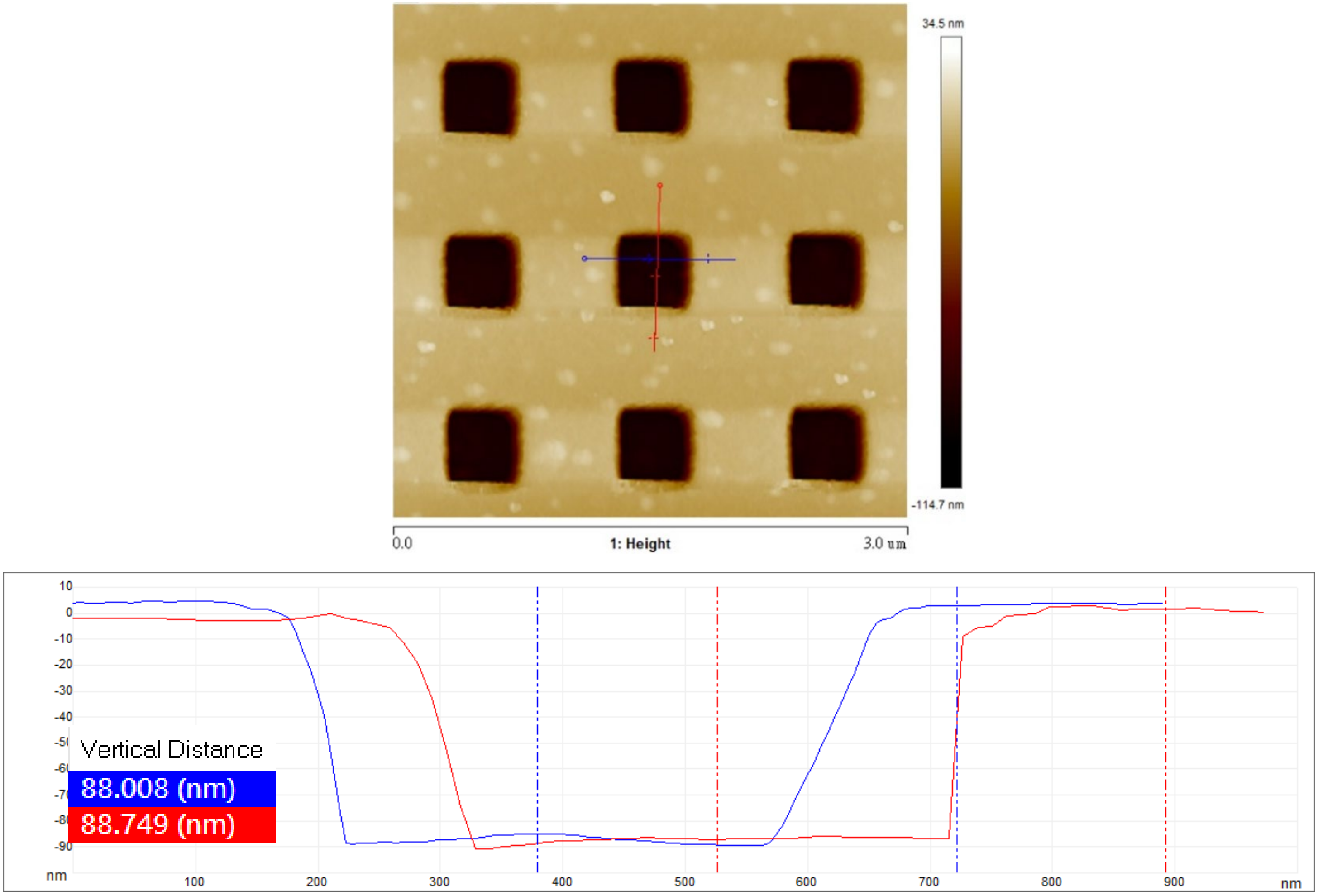

Figure S1. AFM image and cross-sectional profiles along blue and red lines of few-layer MoS_2 /Si NSHs.

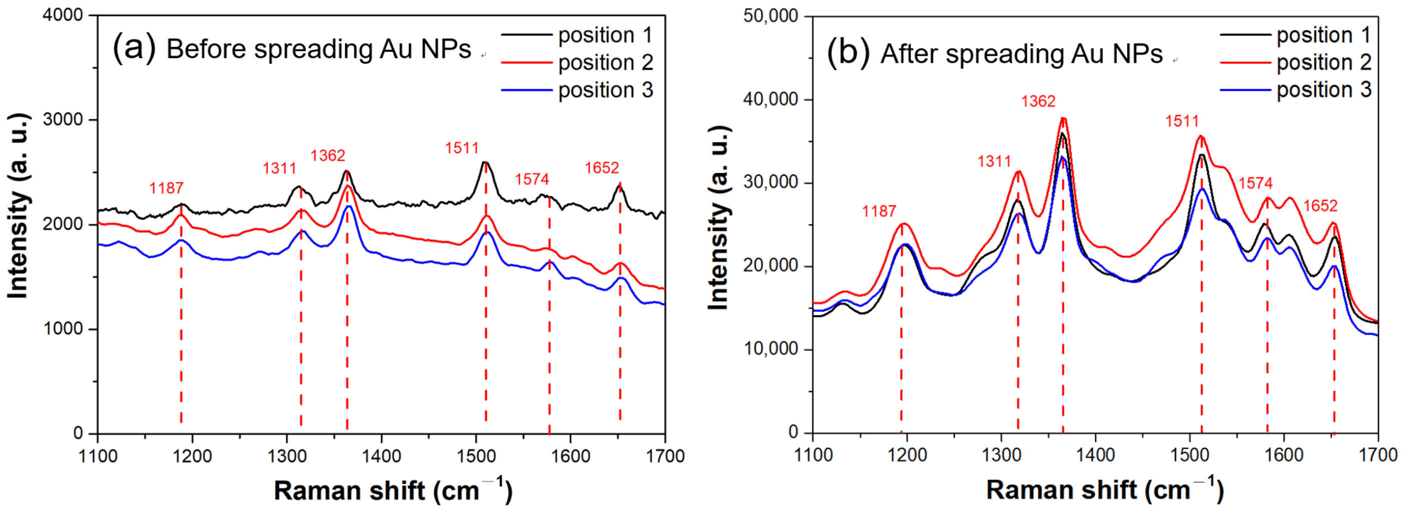

Figure S2. (**a**) Before and (**b**) after spreading Au NPs, SERS signals of R6G molecules (10^{-6} M) from randomly different positions 1–3 on few-layer MoS_2/ Si NSHs.
